# Markers of Kidney Injury: Proenkephalin A and Uromodulin, but Not Dickkopf-3, Are Elevated in Patients After Hematopoietic Stem Cell Transplantation

**DOI:** 10.3390/ijms26083581

**Published:** 2025-04-10

**Authors:** Aleksandra Kaszyńska, Małgorzata Kępska-Dzilińska, Ewa Karakulska-Prystupiuk, Agnieszka Tomaszewska, Grzegorz Władysław Basak, Marcin Żórawski, Zuzanna Jakubowska, Jolanta Małyszko

**Affiliations:** 1 Department of Nephrology, Dialysis and Internal Medicine, Medical University of Warsaw, Banacha 1A, 02-097 Warsaw, Poland; ola.kaszynska@gmail.com (A.K.); malgorzata.kepska@gmail.com (M.K.-D.); zuzanna.jakubowska@wum.edu.pl (Z.J.); 2 Department of Hematology, Transplantation and Internal Medicine, Medical University of Warsaw, Banacha 1A, 02-097 Warsaw, Poland; ewa.prystupiuk@uckwum.pl (E.K.-P.); agnieszka.tomaszewska@wum.edu.pl (A.T.); grzegorz.basak@wum.edu.pl (G.W.B.); 3 Department of Clinical Medicine, Medical University, Szpitalna 37, 15-254 Bialystok, Poland; mzorawski@wp.pl

**Keywords:** hematopoietic stem cell transplantation, acute kidney injury, biomarkers, chronic kidney disease

## Abstract

Kidney injury encompasses a broad spectrum of structural and functional abnormalities, directly associated with stem cell transplantation. Acute kidney injury and chronic kidney disease represent perilous complications of hematopoietic stem cell transplantation (HSCT), with an elevated risk of mortality and progression to end-stage renal disease. The early detection of these complications is, therefore, paramount, and research is increasingly focused on the identification of novel biomarkers of kidney damage. Recently, proenkephalin (PENK), a monomeric peptide that is freely filtered by the glomerulus and thus reflects glomerular filtration very well, has been shown to be an additional useful predictor of the occurrence of acute kidney injury and heart failure. Dickkopf-3 (DKK3) is a glycoprotein secreted by the renal tubular epithelium in response to stress and has been implicated in the development of interstitial fibrosis. It has therefore been evaluated primarily as a marker of fibrosis in chronic kidney disease (CKD), but may also help predict the development of acute kiney injury. Uromodulin is regarded as a renal marker. Previous studies have examined the potential of PENK, DKK-3 and uromodulin as a biomarker in individuals with preserved renal function. However, the urinary levels of PENK, DKK-3 and uromodulin in patients following HSCT have not yet been established. The objective of the present study was to assess urinary PENK, DKK-3, and uromodulin concentrations in patients who had been under ambulatory care of the Hematology, Transplantation and Internal Medicine Department for a minimum of three months following HSCT, and to investigate their correlations with kidney function, as reflected by serum creatinine and eGFR. The study population comprised 80 patients who had undergone allogeneic HSCT for various reasons, primarily hematological malignancies such as acute leukemias and lymphomas. In addition, 32 healthy volunteers were included in order to establish normal ranges for the biomarkers of interest. Urine concentrations of proenkephalin, DKK-3, and uromodulin were evaluated using a commercially available sandwich ELISA immunoassay. Demographic and clinical data were retrieved from the patients’ records. Statistical analyses were conducted using XLSLAT 2022 (Lumivero, Denver, CO, USA) and STATISTICAv13.0 (StatSoft, Tulsa, OH, USA). The results showed that PENK and DKK-3 levels were significantly higher in patients after HSCT compared to healthy volunteers. Furthermore, when patients were divided according to kidney function (below and over 60 mL/min/1.72 m^2^), it was found that the concentration of PENK and DKK-3 were significantly higher in 23 patients with CKD stage 3 relative to patients with eGFR over 60 mL min 1.72 m^2^. In univariate correlations, PENK demonstrated an inverse relationship with eGFR (r: −0.21, *p* < 0.05), while DKK-3 exhibited no significant correlation with creatinine or eGFR.Patients following allogeneic HSCT, despite having normal or near-normal kidney function, exhibited evidence of kidney injury. However, further research is necessary to ascertain the clinical utility of the novel biomarker.

## 1. Introduction

In 1957, E. Donnella Thomas performed the world’s first successful transplantation of hematopoietic stem cells [1]. Since then, the procedure has been systematically improved and has now become a common life-saving method. Currently, approximately 60,000 bone marrow transplants are performed worldwide each year for hematological diseases [2].

The bone marrow transplant procedure involves the infusion of healthy bone marrow stem cells and immune cells to replace diseased cells and cure malignant tumors or hematological disorders. The procedure is preceded by conditioning, i.e., intensive chemotherapy or chemoradiotherapy [3,4]. Three types of conditioning are use: myeloablative (MA) conditioning, reduced-intensity conditioning (RIC), and nonmyeloablative (NMA). The goal of myeloablative conditioning is to completely destroy the recipient's bone marrow, with the intention of destroying the diseases developing in the bone marrow. Myeloablation is achieved in the case of acute myeloid leukemias, usually with busulfan and cyclosporine, which have established efficacy. Unfortunately, the cost of this therapy is significant toxicity, which is why competitive non-myeloablative strategies and techniques are sought. In the non-myeloablative procedure, lower doses of cytostatics are used, which are tolerated by older patients and those with comorbidities, and the final effect of the engraftment is achieved thanks to appropriately applied immunosuppression. Bone marrow transplants can be distinguished: autologous—in which case the stem cells are collected directly from the recipient, and allogeneic—which means that the cells are collected from another person [5]. Stem cells are currently most often collected from peripheral blood by apheresis. During the apheresis procedure, stem cells mobilized after the administration of granulocyte growth factor are collected. Transplantation of cells collected from peripheral blood is associated with faster reconstitution and a lower percentage of graft rejection [6]. In certain cases (e.g., non-cancerous conditions), stem cells are collected directly from the donor's bone marrow because transplantation of such cells is associated with a lower rate of graft-versus-host disease (GVHD) [7].

Thanks to modern treatment, the survival rate of patients after HSCT has increased significantly, but they are at risk of numerous complications. Kidney injury directly associated with stem cell transplantation includes a wide range of structural and functional abnormalities and is associated with increased morbidity and mortality. One of these complications is chronic kidney disease and acute kidney injury, which can lead to end-stage kidney failure. Chronic kidney disease may be secondary to chemotherapy and radiotherapy after transplant, including toxic conditioning and complications.

Acute kidney injury (AKI) occurs commonly after stem cell transplant, affecting 10–73% of patients [8]. The risk of AKI after HSCT depends upon the type of transplantation and conditioning regimen. Transplantation procedures affect both epidemiology and kidney function outcomes. High dose chemotherapy together with total body irradiation bear the higher risk of nausea, vomiting and diarrhea, leading to increased prevalence of prerenal AKI. On the other hand, sepsis, drug-induced interstitial nephritis are risk factors for renal AKI. Postrenal AKI may be due to viral infections, including adeno-polyomaviruses causing hemorrhagic cystitis. The incidence of AKI in allogeneic HSCT is much higher than after autologous stem cell transplantation as GVHD may contribute to the development of AKI following the former [8].

More importantly, AKI is associated with substantial morbidity, including the need for kidney replacement therapy in approximately 5% of patients and the development of chronic kidney disease in up to 60% of transplant recipients [9]. Therefore, prevention, early recognition, and prompt treatment of kidney injury are essential to improving kidney and patient outcomes after HSCT.

Another complication after stem cell transplantation is chronic kidney disease (CKD). It is reported that approximately 20% of patients develop CKD 6–12 months after allogeneic HSCT [8]. CKD after HSCT may be due to several risk factors such as administration of use nephrotoxic drugs including cytostatics, e.g., fludarabine, etoposide, antifungals i.e., amphotericin B, and calcineurin inhibitors) [8,10]. Being older and female are also risk for development of CKD after HSCT [8,10].

Late worsening of kidney function may be also due to the development of radiation found in approximately 20 % of HSCT recipients. It is due to the fact that radiation leads to the injury of vascular endothelium and hemolysis [8,10]. The most common late complication of HSCT is chronic graft versus host disease (cGVHD, chronic graft versus host disease), leading to the development and then progression of CKD. It should be also underlined that glomerulopathy and nephrotic syndrome represent rare symptoms of non-classic cGVHD. Data on these complications are very limited [8]. Moreover, etiology and pathogenesis of nephrotic syndrome in patients with cGVHD after HSCT are still obscure. Membranous nephropathy and minimal change disease are the most prevalent causes of nephrotic syndrome after HSCT, which develops approximately 2 years after stem cell transplantation [8].

The importance of creatinine concentration as the main marker of acute kidney injury is increasingly questioned in many publications [11]. Patients with large muscle mass may initially have higher creatinine concentrations despite normal glomerular filtration.

Removal of part of the kidney may also not cause an increase in creatinine concentration, despite the loss of some nephrons. Therefore, efforts are constantly being made to achieve broader and more accurate diagnostics for early detection of acute kidney injury. Currently, several biomarkers of acute kidney injury have been characterized. Molecules that can be detected in urine or blood confirm or exclude structural kidney damage. Currently, they are only clinically used to support the diagnosis of AKI, but there is great hope that in the future they will replace the determination of creatinine as the main marker for diagnosing AKI. Specific biomarkers in blood and urine are released by various mechanisms during renal tubule damage [12]. It is important that the specific biomarker comes from the damaged kidney and reflects a process closely related to the damage to the given tissue [13].

In recent years, several new biomarkers have been proposed to fulfill this unmet medical need in the accurate determination of kidney function [14].

PENK, a monomeric peptide (approximately 4.5 kDa), is a byproduct of the cleaving of enkephalins of preproenkephalin A and appears to be freely filtered through the glomerulus; thus, it might become a functional biomarker for kidney function [15], implying possible direct effects on kidney function. The relatively small size of PENK (4.5 kDa) makes free filtration through the glomerulus plausible, and combined with its longer half-life, PENK could be a new functional biomarker for GFR. PENK A has been suggested as a novel biomarker for AKI in perioperative and critical settings [16,17,18].

In the recent studies Dicklopf3 protein-Dkk3 was suggested to serve as the clinical biomarker of renal damage and a prognostic biomarker for estimating the CKD risk progression. Urinary Dkk3 was an indicator of tubular injury and predictor of both short-term glomerular filtration rate (GFR) loss as well as the long-term CKD progression, independent from albuminuria or the underlying cause of CKD in humans [19,20,21]. It was also demonstrated that elevated urinary Dkk3 level was associated with an increased risk for AKI and CKD following cardiac surgery [20,21].

The predominant protein in urine of healthy people is uromodulin, also known as Tamm-Horsfall protein [22]. However, many studies in recent years have shown that both urinary and serum uromodulin correlate with renal function, graft survival, cardiovascular disease, glucose metabolism and mortality from all causes. Serum uromodulin correlates with nephron mass, and is elevated in kidney transplant recipients in relation to initial graft function [23]. It was reported that serum uromodulin was more sensitive in detecting of early stages of CKD than serum creatinine, and cystatin C considered conventional markers [24,25]. It was also suggested that uromodulin could serve as early biomarker of fibrotic and atrophic renal injury [26].

The present study aimed to assess the presence of biomarkers of kidney injury in patients who have undergone allogeneic HSCT at least three months prior to demonstrate that patients who have received such a transplant, and who demonstrate normal or near-normal kidney function, exhibit indications of kidney injury. The potential causes of this injury may include comorbidities, prior chemotherapy, conditioning regimens, complement activation, calcineurin therapy following HSCT, or the administration of other nephrotoxic medications.

## 2. Results

**Figure 1:**
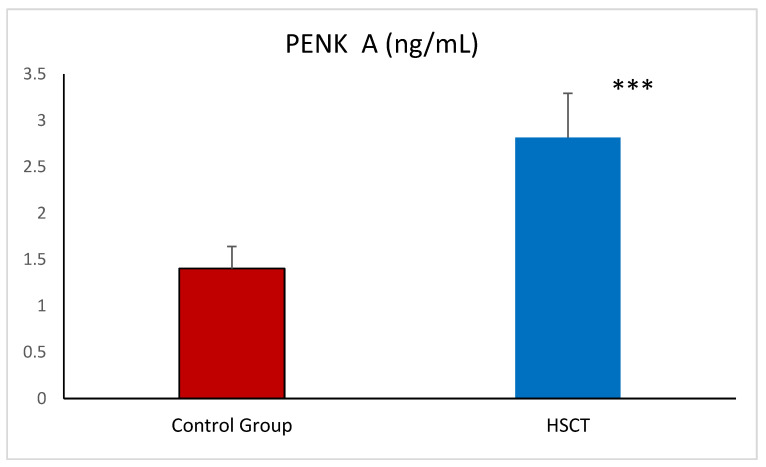
Proenkephalin A—PENK A in the control group and patients after HSCT—hematopoietic stem cell transplantation. Data given are means ± SD. *** *p* < 0.001.

**Figure 2:**
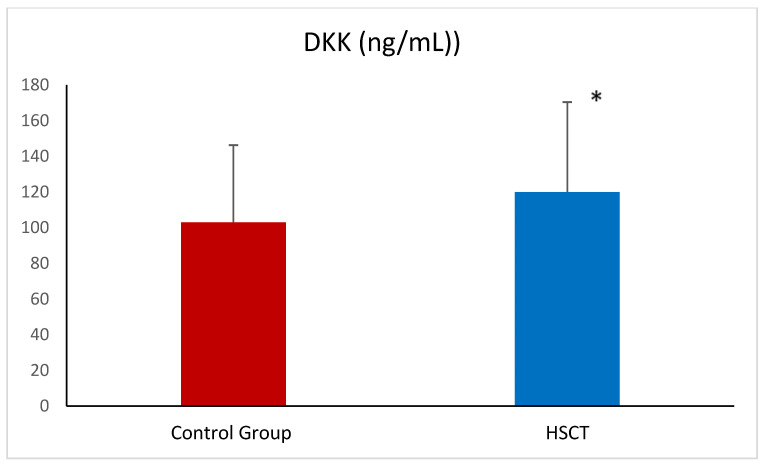
Dickkopf-3 (DKK-3) in the control group and patients after HSCT—hematopoietic stem cell transplantation. Data given are means ± SD. * *p* < 0.05.

PENK was significantly higher in patients after HSCT when compared to healthy volunteers (Figure 1). In addition, CKD was present in 29% of the patients after HSCT (23 out of 80). When we divided patients according to kidney function (below and over 60 mL/min/1.72 m^2^), we found that concentration of PENK was significantly higher in 23 patients with CKD stage 3 relative to patients with eGFR over 60 mL/min/1.72 m^2^. The level of DKK-3 was also significantly higher in patients after HSCT compared to healthy volunteers (Figure 2).

**Figure 3:**
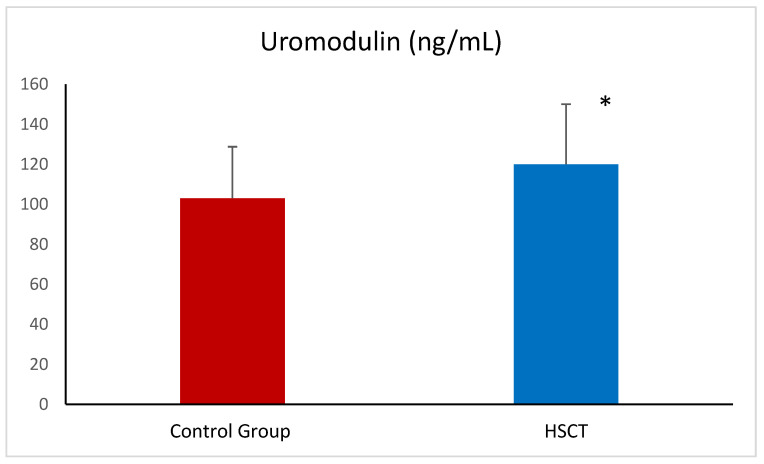
Uromodulin in the control group and patients after HSCT—hematopoietic stem cell transplantation. Data given are means ± SD. * *p* < 0.05.

**Figure 4:**
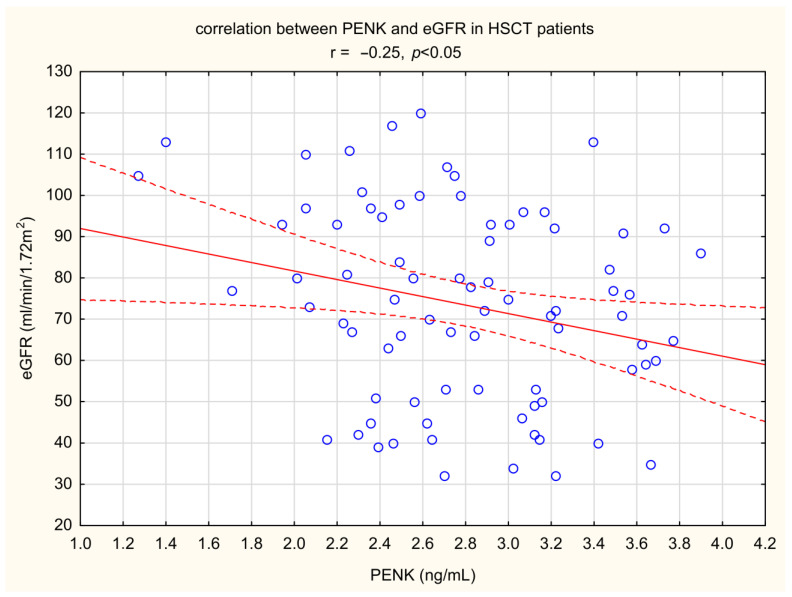
Correlation between PENK A (proenkephalin A) and estimated glomerular filtration rate—eGFR patients after HSCT—hematopoietic stem cell transplantation (shown with blue circles, solid line—mean, dashed lines—coefficient interval).

The level of uromodulin was also significantly higher in patients after HSCT compared to healthy volunteers (Figure 3). In univariate correlations PENK was inversely related to eGFR (r= −0.21, *p* < 0.05) (Figure 4). No statistically significant correlations were found between DKK-3 and serum creatinine or eGFR. Uromodulin was also not significantly related to either serum creatinine or eGFR.

## 3. Discussion

Hematopoietic stem cell transplantation (HSCT) is the most effective therapy for the treatment of a variety of hematological disorders, but is associated with a high risk of organ toxicity.

In recent years, the topic of kidney injury in patients after bone marrow transplantation has been discussed more and more often. Acute kidney injury predisposes to relapses of the underlying disease, may cause chronic kidney disease, end-stage renal failure requiring dialysis therapy. Thus, it may lead to an increased number of hospitalizations and mortality [26]. Diagnosis of acute kidney injury is associated with the risk of cardiovascular diseases, stroke, hypertension, osteodystrophy [27,28].

Due to the above, studies are increasingly being conducted to prevent AKI and early implementation of treatment to prevent the development of short- and long-term complications. Studies have confirmed that improvement in kidney function within 48 to 72 h is associated with better results in long-term follow-up, compared to patients with acute kidney injury lasting more than 72 h [29].

It should be emphasized that so far scientists have not defined complete or partial kidney regeneration after an episode of AKI in patients after HSCT [26,30]. In patients after HSCT, the incidence of AKI is approximately 55.1%, including severe stage III AKI in 8.3% [31]. It has been confirmed that patients who have had severe acute kidney injury requiring renal replacement therapy have an increased risk of recurrence of AKI [31]. AKI in patients after bone marrow transplantation (both allogeneic and autologous transplantation) occurs mainly in the first 30 days after the procedure. During this period, a higher risk of developing AKI has been confirmed due to toxicity associated with conditioning regimens, as well as the risk of developing sepsis and drug-induced kidney damage [32]. GVHD disease is also a risk factor for AKI, but it should be emphasized that this risk occurs more than 100 days after alloHSCT [8]

CKD is diagnosed in approximately 20% of patients six to twelve months after HSCT [10]. It should be emphasized that CKD after HSCT has a multifactorial etiology [10]. CKD is a serious long-term complication of HCT and is associated with a history of AKI [8]. Despite normal or near-normal renal function, there is evidence of renal injury after allogeneic HSCT, which may be due to comorbidity, previous chemotherapy, conditioning, complement activation, post-HSCT calcineurin therapy, other possible nephrotoxic drugs, and so on [8]. The greatest decrease in eGFR was observed within the first year after transplantation (eGFR decreased from 98 mL/min/1.73 m^2^ to 78 mL/min/1.73 m^2^ after 1 year). It is worth noting that decreased eGFR was associated with a higher risk of mortality, although the eGFR level was stable after the initial decrease [18]. Our cross-sectional study focused on PENK, a possible biomarker of renal injury, which was significantly higher in both subgroups of patients with eGFR below and above 60 mL/min/1.72 m^2^. These results provide a deeper understanding of this disease and allow earlier detection of kidney damage. To our knowledge, this study is the first to highlight the importance of urinary biomarkers such as PENK A in post-HSCT kidney injury. PENK A has also been reported to reflect glomerular dysfunction in individuals with adequate and reduced filtration rates, as a predictive marker of CKD [18,33]. It was reported previously, that PENK correlated with measured GFR (*R*^2^ = 0.90, *p* < 0.0001) significantly stronger relative to correlation between estimated GFR calculated using the MDRD formula (*p* = 0.06) and endogenous creatinine clearance (*p* = 0.08) [34].

Similar relationship was shown between PENK and GFR estimated using creatinine-based formula or CKD-EPI race free formula [35,36,37]. In kidney transplant recipients with steady renal state and 95 healthy kidney donors, PENK levels were inversely correlated with eGFR (r_s_ = −0.80, *p* < 0.001) [35]. As it was reported recently, PENK is increased in impaired kidney function [38]. It was also stressed that PENK reflected kidney function in patients with and without kidney disease, in critically ill patients independent of sex, age and inflammation [39,40,41].

In one of the recent studies on PENK A, Tichy et al. [42] performed a prospective, observational, single-center study on 61 patients undergoing cardiac surgery for a cardiopulmonary bypass. They investigated whether PENK A could guide successful liberation from continuous kidney replacement therapy. They measured PENK Aat the time of diagnosis of AKI and at the initiation of and liberation from continuous kidney replacement therapy. In the studied group 20 patients had AKI requiring kidney replacement therapy. Then, they compared values of PENK A in the group liberated from kidney replacement therapy *(n* = 11) versus continuing kidney support. (*n* = 9) They found that PENK A levels in patients successfully liberated from kidney support were lower in comparison with PENK A levels in patients unsuccessfully liberated from continuous kidney replacement therapy. Moreover, in this group liberated from kidney support urinary output was significantly higher.

Tichy et al. [42] concluded that PENK Awas an emerging renal biomarker that is promising for monitoring renal function and in additionally renal recovery. Similar data came from the multicenter study performed by van Groote et al. [43]. They showed that low PENK A was associated with an early and successful liberation from continuous kidney replacement therapy in a cohort of critically ill subjects. However, these observations came from a post hoc analysis. Concerning PENK A levels in the study of Tichy et al. [42], they were similar like in other publications [16,43,44]. Bullen et al. [45] in a nested biracial cohort of 4400 participants among the REasons for Geographic and Racial Differences in Stroke (REGARDS) in Unites States, searched for the association of PENK A with incident chronic kidney disease and other kidney outcomes. They found that PENK A was associated with CKD progression in incident albuminuria in non-diabetic patients, whereas no associations were found between incident CKD and PENK A.

Finally, we discussed the role of PENK in perioperative and intensive care setting, as an emerging biomarker, as well as in chronic kidney disease [46,47].

While most studies have been focused on investigating PENK A in individuals with AKI or CKD, PENK A levels and its determinants in patients after HSCT were not assessed. Since patients with CKD and AKI, commonly found after HSCT are susceptible to a rapid decline in kidney function requiring even dialysis treatment, further validation of the role of PENK A as an indicator of GFR is required in this setting. Assessment of PENK A may offer the hope of a quick and efficient assessment of renal damage after HSCT and avoiding the development of CKD. Shi et al. [48] published a systematic review and meta-analysis on biomarkers of persistent acute kidney injury in 2025. They screened 31 studies from 2356 studies from various databases including Medline, PubMed, Embase, and Cochrane and evaluated the diagnostic value of 7 biomarkers for persistent acute kidney injury i.e., urinary C–C motif chemokine ligand 14 (CCL14), tissue inhibitor of metalloproteinase-2 and insulin-like growth factor-binding protein-7 (TIMP-2 and IGFBP7), neutrophil gelatinase-associated lipocalin (NGAL), plasma cystatin C (pCysC), soluble urokinase plasminogen activator receptor (suPAR), proenkephalin (PENK A) and urinary dickkopf-3:urinary creatinine (uDKK3:uCr). They found that CCL14 had the best diagnostic efficacy. On the other hand, PENK A, uDKK3:uCr, and suPAR were not included in the meta-analysis due to a limited number of studies.

DKK3 as a stress-induced, renal tubular epithelial cell-derived, pro-fibrotic molecule. It is a glycoprotein, which is expressed in the proximal tubules, and that is increased in patients with declining eGFR compared to patients with stable disease [49]. Recently data suggest that urinary DKK3 is a sensitive indicator of ongoing tubular injury independent of eGFR and albuminuria [19,20,49]. In addition, it decreases in the period of time patients have stable disease [49]. DKK3 levels are correlated with serum creatinine and eGFR in patients with CKD [50], which makes it a promising marker to monitor progression of disease. Moreover, DKK-3 was tested in different settings, in particular in cardiac surgery and contrast media induced acute kidney injury, when time is kidney insult is known [51]. It was concluded that urinary DKK3 was an independent predictor of contrast-induced-AKI even in the absence of overt chronic kidney disease [52]. Moreover, preoperative urinary DKK3 is an independent predictor for postoperative AKI and for subsequent loss of kidney function [50]. It was suggested that DKK-3 may serve as a potential predictor for AKI [49] and increased urinary DKK3 levels identified patients at high risk for short-term CKD progression, regardless of the cause of kidney disease, baseline kidney function and albuminuria [50]. In addition, urinary DKK3 was also increased in 118 patients with biopsy proven diabetic nephropathy with normal kidney function (GFR ≥ 90 mL/min/1.73 m^2^) in cross-sectional study by González et al. [53]. Interstitial fibrosis on kidney biopsy correlated positively with DKK3, age, proteinuria, as well as monocyte chemoattractant protein-1 (MCP-1) and liver-type fatty acid-binding protein (L-FABP). More interestingly, age, proteinuria, MCP-1, DKK3 and epidermal growth factor (EGF), when included in multivariate the model, predict interstitial fibrosis surface, accounting for 59% of the variability.

Singh et al. [54] in their review paper discussed performance of several AKI biomarkers, including PENK and DKK-3 in surgical settings as well as their application to guide patients’ postoperative care. They described a variety of candidates potentially allowing the earlier identification of AKI and thereby allow timely intervention. According to them, presence of albuminuria, suPAR and DKK3 enhance the risk prediction of AKI in postoperative setting. Xing et al. [55] performed a systematic review and meta-analysis on the role of urinary DKK3 and prediction of AKI. They screened English databases (PubMed, Embase, Cochrane, and WOS) and Chinese databases (VIP, WanFang data, and China National Knowledge Internet) for this purpose. Finally, they included a total of 5 studies involving 2787 patients, 4 of them focused on contrast-induced acute kidney injury (CI-AKI) and the remaining one focused on AKI associated with cardiac surgery. They demonstrated that urine DKK3 had high diagnostic accuracy for AKI, however, due to small number of available studies for analysis, subgroup analysis was not performed. They advocated for large sample size clinical studies to validate these findings.

Uromodulin as an early biomarker of fibrotic and atrophic kidney damage [25], serum uromodulin could be a valuable tool for the early detection and monitoring of kidney-related pathologies. In the Cardiovascular Health Study, serum uromodulin was found to be more strongly associated with eGFR than was urinary uromodulin. In a separate small cohort of 112 healthy living donors, there was no correlation between serum uromodulin levels, measured glomerular filtration rate (mGFR) and eGFR, in contrast to findings in patients with CKD [56]. Low urinary uromodulin levels have been shown to predict the development of CKD over 9 to 10 years of follow-up [57], and the rapid decline in renal function in CKD patients leads to progression to end-stage renal disease within 1 year [58]. In two independent cohorts of the Framingham Heart Study and the Atherosclerosis Risk in Communities Study, which focused on the incidence of CKD, elevated urinary uromodulin concentrations preceded the onset of CKD [59,60].

In the recent study in the animal model of acute kidney injury Vonbrunn et al. [61] showed a transient rise in serum uromodulin in parallel with loss of uromodulin-positive cells. They suggested that it was due to a temporary release of uromodulin from destroyed tubular cells into the blood. They used the model of ischemia/reperfusion for AKI and model of uninephrectomy and 5/6 nephrectomy for CKD. They also concluded that rise in serum uromodulin appears not as a marker of chronic kidney failure, but also reflect acute loss of functional and cellular integrity of kidney epithelia in acute setting.

We fully recognize that our study is limited. Patients after hematopoietic stem cell transplantation represent a very heterogeneous group of patients and it is therefore likely that the results could have been influenced by a variety of confounding factors. The study was single-center. Therefore, the results may not be generalizable Moreover, we acknowledge limitations related to external validity and data availability. We would like to stress that we evaluated patients at least 100 days after HSCT to exclude the effects of conditioning regimen, AKI or nephrotoxic drugs i.e., gentamicin, amikacin, vancomycin, amphotericin B, and foscarnet used in the early posttransplant period.

As data of our study on biomarkers after HSCT was accepted as the oral presentation during The Transplantation Congress in Istanbul, Türkiye 22–25 September 2024 and abstract was published in Transplantation [62]. During the oral presentation discussion was so vivid and fruitful that prompted us to share our results in a form of full paper.

## 4. Materials and Methods

A study was conducted on 80 prevalent patients (40 females) who underwent allogeneic HSCT treatment in our center between 1998 and 2020 due to hematological pathologies (acute myeloid leukemia, *n* = 34, acute lymphoblastic leukemia, *n* = 18, lymphoma *n* = 8, other, i.e., myelodysplastic syndrome, *n* = 5, chronic myelomonocytic leukemia, *n* = 4, chronic lymphocytic leukemia, *n* = 4,aplastic anemia, *n* = 3, primary myelofibrosis, *n* = 2, AL amyloidosis, *n* = 1, Waldenstrom macroglobulinemia, *n* = 1) and a control group of 32 healthy volunteers (16 females) to obtain normal ranges for biomarkers. HSCT from a related donor was performed in 13 patients, while the remaining 67 patients underwent allogeneic HSCT. The clinical and biochemical data are presented below in Table 1. In the Table 2 some clinical and biochemical parameters in HSCT recipients in relation to CKD stage.

CKD was defined according to KDIGO [63] as a set of abnormalities in the function or structure of the kidneys characterized by symptoms lasting for a period of three months or more, in which the glomerular filtration rate (GFR) is less than 60 mL/min/1.73 m^2^ or greater, but with evidence of damage to the kidney structure. The main indicators of kidney damage are: albuminuria (presence of more than 30 mg of albumin in a 24-h urine collection or 30 mg/g of albumin in an isolated urine sample corrected for urine creatinine concentration), changes in imaging studies, hematuria, leukocyturia, histopathological changes found in kidney biopsy, status after kidney transplantation [63]. GFR was estimated using CKD-EPI formula based on creatinine [63].

The mean age of patients with acute leukemia was 48 years (including acute myeloid leukemia 52 years, acute lymphoblastic leukemia 38 years). The mean age of lymphoma patients was 46 years.

In this cross-sectional study PENK A, a biomarker of kidney injury in urine was evaluated using commercially available assay (Human Proenkephalin (PENK) ELISA kit) from SunRedBio, Shanghai, China. Dkk3 level was assayed using Human Dickkopf 3 ELISA kit (Bioassay Technology Laboratory, Shanghai, China, Cat. No. E2065Hu). Uromodulin was assesses using human uromodulin (THP/UMOD) ELISA kit (Bioassay Technology Laboratory, Shanghai, China, (Catalogue No: E4743HU), according to the manufacturer’s instructions.

## 5. Conclusions

HSCT become a cornerstone therapy for many patients with hematological diseases, however, it is associated with several adverse events including nephrotoxicity. The prevalence of CKD reaches almost 30% in this population. Specific biomarkers may be useful in further planning of the therapy, appropriate chemotherapy selection, as well as type of conditioning regimen and immunosuppressive protocol after stem cell transplantation. In patients after HSCT the early diagnosis of kidney failure is of utmost importance as it is associated with worse outcomes. Nephroprotective strategies are crucial to prevent development of CKD, a risk factor for increased morbidity and mortality.

## Figures and Tables

**Table 1:** Some clinical and biochemical parameters in HSCT recipients.

	Control Group	HSCT
Age (years)	49 ± 10	48 ± 14
Time after transplantation (months)	NA	103.3 ± 50.5
Hemoglobin (g/dL)	13.87 ± 0.89	12.87 ± 1.99
Acute myeloid leukemia (%)	NA	43
serum creatinine (mg/dL)	0.86 ± 0.35	1.1 ± 0.37 *
eGFR by CKD-EPI (mL/min/1.72 m^2^)	96.9 ± 13.0	73.5 ± 23.5 **
Erythrocyturia (%)	0	11
Leukocyturia (%)	0	6.25

eGFR—estimated glomerular filtration rate; CKD-EPI chronic kidney disease-epidemiological collaboration; * *p* < 0.05, ** *p* < 0.01, NA—not available.

**Table 2:** Some clinical and biochemical parameters in HSCT recipients in relation to CKD stage.

	HSCT with eGFR > 60 (mL/min/1.72 m^2^)	HSCT with eGFR < 60 (mL/min/1.72 m^2^)
Age (years)	46 ± 10	50 ± 17
Time after transplantation (months)	87 ± 43	119 ± 52 *
Hemoglobin (g/dL)	13.01 ± 0.91	12.03 ± 1.01 *
Acute myeloid leukemia	27	7 **
Acute lymphocytic leukemia	10	8
Lymphoma	4	4
serum creatinine (mg/dL)	0.95 ± 0.21	1.55 ± 0.30 **
eGFR by CKD-EPI (ml/min/1.72 m^2^)	85.28 ± 16.42	46.10 ± 7.80 **
Erythrocyturia (%)	0	11
Leukocyturia (%)	0	6.25
PENK A (ng/mL)	2.57 ± 0.43	2.90 ± 0.44 *
DKK-3 (ng/mL))	47.69 ± 13.25	49.15 ± 14.65
Uromodulin (ng/mL)	101.77 ± 28.12	119.70 ± 27.73 *

HSCT—hematopoietic stem cell transplantation; PENK A—proenkephalin A; DKK-3—dickkopf-3; * *p* < 0.05, ** *p* < 0.01.

## Data Availability

The data presented in this study are available on request from the corresponding author.

## References

[1] Thomas E.D., Lochte H.L., Lu W.C., Ferrebee J.W. (1957). Intravenous infusion of bone marrow in patients receiving radiation and chemotherapy. N. Engl. J. Med..

[2] Appelbaum F.R. (2007). Hematopoietic-cell transplantation at 50. N. Engl. J. Med..

[3] World Health Organization (WHO) (2018). Haematopoietic Stem Cell Transplantation HSCtx.

[4] Bazinet A., Popradi G. (2019). A general practitioner’s guide to hematopoietic stem-cell transplantation. Curr. Oncol..

[5] Thomas E.D., Buckner C.D., Banaji M., Clift R.A., Fefer A., Flournoy N., Goodell B.W., Hickman R.O., Lerner K.G., Neiman P.E. (1977). One hundred patients with acute leukemia treated by chemotherapy, total body irradiation, and allogeneic marrow transplantation. Blood.

[6] Gertz M.A. (2010). Current status of stem cell mobilization. Br. J. Haematol..

[7] Stem Cell Trialists’ Collaborative Group (2005). Allogeneic peripheral blood stem-cell compared with bone marrow transplantation in the management of hematologic malignancies: An individual patient data meta-analysis of nine randomized trials. J. Clin. Oncol..

[8] Renaghan A.D., Jaimes E.A., Malyszko J., Perazella M.A., Sprangers B., Rosner M.H. (2020). Acute Kidney Injury and CKD Associated with Hematopoietic Stem Cell Transplantation. Clin. J. Am. Soc. Nephrol..

[9] Kępska-Dzilińska M., Zhymaila A., Malyszko J. (2022). Kidney damage in patients after allogeneic stem cell transplantation. Wiad Lek.

[10] Saddadi F., Hakemi M., Najafi I., Moghadam K., Ghavamzadeh A., Jahani M., Ganji M., Amini M., Soleimanian T. (2009). Chronic kidney disease after hematopoietic cell transplantation: Frequency, risk factors, and outcomes. Transpl. Proc..

[11] Bennett M., Dent C.L., Ma Q., Dastrala S., Grenier F., Workman R., Syed H., Ali S., Barasch J., Devarajan P. (2008). Urine NGAL predicts severity of acute kidney injury after cardiac surgery: A prospective study. Clin. J. Am. Soc. Nephrol..

[12] Wen Y., Parikh C.R. (2021). Current concepts and advances in biomarkers of acute kidney injury. Crit. Rev. Clin. Lab. Sci..

[13] Schrezenmeier E.V., Barasch J., Budde K., Westhoff T., Schmidt-Ott K.M. (2017). Biomarkers in acute kidney injury—Pathophysiological basis and clinical performance. Acta Physiol..

[14] Endre Z.H. (2018). Assessing renal recovery after acute kidney injury: Can biomarkers help?. Nephron.

[15] Denning G.M., Ackermann L.W., Barna T.J., Armstrong J.G., Stoll L.L., Weintraub N.L., Dickson E.W. (2008). Proenkephalin expression and enkephalin release are widely observed in non-neuronal tissues. Peptides.

[16] Shah K.S., Taub P., Patel M., Rehfeldt M., Struck J., Clopton P., Mehta R.L., Maisel A.S. (2015). Proenkephalin predicts acute kidney injury in cardiac surgery patients. Clin. Nephrol..

[17] Marino R., Struck J., Hartmann O., Maisel A.S., Rehfeldt M., Magrini L., Melander O., Bergmann A., Di Somma S. (2015). Diagnostic and short-term prognostic utility of plasma pro-enkephalin (pro-ENK) for acute kidney injury in patients admitted with sepsis in the emergency department. J. Nephrol..

[18] Schulz C.-A., Christensson A., Ericson U., Almgren P., Hindy G., Nilsson P.M., Struck J., Bergmann A., Melander O., Orho-Melander M. (2017). High level of fasting plasma proenkephalin-A predicts deterioration of kidney function and incidence of CKD. J. Am. Soc. Nephrol..

[19] Zewinger S., Rauen T., Rudnicki M., Federico G., Wagner M., Triem S., Schunk S.J., Petrakis I., Schmit D., Wagenpfeil S. (2018). Dickkopf-3 (DKK3) in Urine Identifies Patients with Short-Term Risk of eGFR Loss. J. Am. Soc. Nephrol..

[20] Schunk S.J., Speer T., Petrakis I., Fliser D. (2021). Dickkopf 3-a novel biomarker of the ‘kidney injury continuum’. Nephrol. Dial. Transpl..

[21] Schunk S.J., Floege J., Fliser D., Speer T. (2021). WNT–β-catenin signaling—A versatile player in kidney injury and repair. Nature.

[22] Dawnay A.B., Cattell W.R. (1981). Serum Tamm-Horsfall Glycoprotein Levels in Health and in Renal Disease. Clin. Nephrol..

[23] Usui R., Ogawa T., Takahashi H., Iwasaki C., Koike M., Morito T., Hatano M., Nitta K. (2021). Serum Uromodulin Is a Novel Renal Function Marker in the Japanese Population. Clin. Exp. Nephrol..

[24] Steubl D., Block M., Herbst V., Nockher W.A., Schlumberger W., Satanovskij R., Angermann S., Hasenau A.-L., Stecher L., Heemann U. (2016). Plasma Uromodulin Correlates with Kidney Function and Identifies Early Stages in Chronic Kidney Disease Patients. Medicine.

[25] Smirnov A.V., Khasun M., Kayukov I.G., Galkina O.V., Sipovski V.G., Parastaeva M.M., Bogdanova E.O. (2018). Serum Uromodulin as an Early Biomarker of Tubular Atrophy and Interstitial Fibrosis in Patients with Glomerulopathies. Ter. Arkhiv.

[26] Forni L.G., Darmon M., Ostermann M., Oudemans-van Straaten H.M., Pettilä V., Prowle J.R., Schetz M., Joannidis M. (2017). Renal recovery after acute kidney injury. Intensive Care Med..

[27] Kellum J.A., Sileanu F.E., Bihorac A., Hoste E.A., Chawla L.S. (2017). Recovery after acute kidney injury. Am. J. Respir. Crit. Care Med..

[28] Thongprayoon C., Hansrivijit P., Kovvuru K., Kanduri S.R., Torres-Ortiz A., Acharya P., Gonzalez-Suarez M.L., Kaewput W., Bathini T., Cheungpasitporn W. (2020). Diagnostics, risk factors, treatment and outcomes of acute kidney injury in a new paradigm. J. Clin. Med..

[29] Coca S.G., King J.T., Rosenthal R.A., Perkal M.F., Parikh C.R. (2010). The duration of postoperative acute kidney injury is an additional parameter predicting long-term survival in diabetic veterans. Kidney Int..

[30] Cerdá J., Liu K.D., Cruz D.N., Jaber B.L., Koyner J.L., Heung M., Okusa M.D., Faubel S. (2015). Promoting kidney function recovery in patients with AKI requiring RRT. Clin. J. Am. Soc. Nephrol..

[31] Kanduri S.R., Cheungpasitporn W., Thongprayoon C., Bathini T., Kovvuru K., Garla V., Medaura J., Vaitla P., Kashani K.B. (2020). Incidence and mortality of acute kidney injury in patients undergoing hematopoietic stem cell transplantation: A systematic review and meta-analysis. QJM Int. J. Med..

[32] Hingorani S. (2016). Renal complications of hematopoietic-cell transplantation. N. Engl. J. Med..

[33] Lima C., Gorab D.L., Fernandes C.R., Macedo E. (2022). Role of proenkephalin in the diagnosis of severe and subclinical acute kidney injury during the perioperative period of liver transplantation. Pract. Lab Med..

[34] Beunders R., van Groenendael R., Leijte G.P., Kox M., Pickkers P. (2020). Proenkephalin Compared to Conventional Methods to Assess Kidney Function in Critically Ill Sepsis Patients. Shock.

[35] Kieneker L.M., Hartmann O., Struck J., Bergmann A., Gansevoort R.T., Joosten M.M., van den Berg E., de Boer R.A., Bakker S.J. (2017). Plasma Proenkephalin and Poor Long-Term Outcome in Renal Transplant Recipients. Transpl. Direct.

[36] Beunders R., Donato L.J., van Groenendael R., Arlt B., Carvalho-Wodarz C., Schulte J., Coolen A.C., Lieske J.C., Meeusen J.W., Jaffe A.S. (2023). Assessing GFR with Proenkephalin. Kidney Int. Rep..

[37] Nilsson C., Christensson A., Nilsson P.M., Melander O., Bennet L. (2021). Pro-Enkephalin and its association with renal function in Middle Eastern immigrants and native Swedes. Scand. J. Clin. Lab. Investig..

[38] Khorashadi M., Beunders R., Pickkers P., Legrand M. (2020). Proenkephalin: A New Biomarker for Glomerular Filtration Rate and Acute Kidney Injury. Nephron.

[39] Smeets N.J.L., Hartmann O., Schulte J., Schreuder M.F., de Wildt S.N. (2022). Proenkephalin A as a marker for glomerular filtration rate in critically ill children: Validation against gold standard iohexol GFR measurements. Clin. Chem. Lab. Med..

[40] Caironi P., Latini R., Struck J., Hartmann O., Bergmann A., Bellato V., Ferraris S., Tognoni G., Pesenti A., Gattinoni L. (2018). Circulating Proenkephalin, Acute Kidney Injury, and Its Improvement in Patients with Severe Sepsis or Shock. Clin. Chem..

[41] Donato L.J., Meeusen J.W., Lieske J.C., Bergmann D., Sparwaßer A., Jaffe A.S. (2018). Analytical performance of an immunoassay to measure proenkephalin. Clin. Biochem..

[42] Tichy J., Hausmann A., Lanzerstorfer J., Ryz S., Wagner L., Lassnigg A., Bernardi M.H. (2024). Prediction of Successful Liberation from Continuous Renal Replacement Therapy Using a Novel Biomarker in Patients with Acute Kidney Injury after Cardiac Surgery-An Observational Trial. Int. J. Mol. Sci..

[43] Von Groote T., Albert F., Meersch M., Koch R., Gerss J., Arlt B., Sadjadi M., Porschen C., Pickkers P., Zarbock A. (2023). Evaluation of Proenkephalin A 119–159 for liberation from renal replacement therapy: An external, multicenter pilot study in critically ill patients with acute kidney injury. Crit. Care.

[44] Dépret F., Hollinger A., Cariou A., Deye N., Vieillard-Baron A., Fournier M.C., Jaber S., Damoisel C., Lu Q., Monnet X. (2020). Incidence and Outcome of Subclinical Acute Kidney Injury Using penKid in Critically Ill Patients. Am. J. Respir. Crit. Care Med..

[45] Bullen A.L., Katz R., Poursadrolah S., Short S.A.P., Long D.L., Cheung K.L., Sharma S., Al-Rousan T., Fregoso A., Schulte J. (2024). Plasma proenkephalin A and incident chronic kidney disease and albuminuria in the REasons for Geographic And Racial Differences in Stroke (REGARDS) cohort. BMC Nephrol..

[46] Walczak-Wieteska P., Zuzda K., Małyszko J., Andruszkiewicz P. (2024). Proenkephalin A 119–159 in Perioperative and Intensive Care-A Promising Biomarker or Merely Another Option?. Diagnostics.

[47] Grycuk W., Jakubowska Z., Malyszko J. (2025). Proenkephalin (PENK): A functional biomarker in chronic kidney diseases—Hope or just a new bystander?. J. Nephrol..

[48] Shi K., Jiang W., Song L., Li X., Zhang C., Li L., Feng Y., Yang J., Wang T., Wang H. (2025). Persistent acute kidney injury biomarkers: A systematic review and meta-analysis. Clin. Chim. Acta.

[49] Canki E., Kho E., Hoenderop J.G.J. (2024). Urinary biomarkers in kidney disease. Clin. Chim. Acta.

[50] Dziamałek-Macioszczyk P., Winiarska A., Pawłowska A., Wojtacha P., Stompór T. (2023). Patterns of Dickkopf-3 Serum and Urine Levels at Different Stages of Chronic Kidney Disease. J. Clin. Med..

[51] Sun Y., Xiao Z., Yang S., Hao C., Zhao H., An Y. (2025). Advances and insights for DKK3 in non-cancerous diseases: A systematic review. PeerJ.

[52] Schunk S.J., Zarbock A., Meersch M., Küllmar M., Kellum J.A., Schmit D., Wagner M., Triem S., Wagenpfeil S., Gröne H.J. (2019). Association between urinary dickkopf-3, acute kidney injury, and subsequent loss of kidney function in patients undergoing cardiac surgery: An observational cohort study. Lancet.

[53] González J., Jatem E., Roig J., Valtierra N., Ostos E., Abó A., Santacana M., García A., Segarra A. (2022). Usefulness of urinary biomarkers to estimate the interstitial fibrosis surface in diabetic nephropathy with normal kidney function. Nephrol. Dial Transpl..

[54] Singh R., Watchorn J.C., Zarbock A., Forni L.G. (2024). Prognostic Biomarkers and AKI: Potential to Enhance the Identification of Post-Operative Patients at Risk of Loss of Renal Function. Res. Rep. Urol..

[55] Xing H., Jiang Z., Wu Y., Ou S., Qin J., Xue L., Wu W. (2023). The role of urinary Dickkopf-3 in the prediction of acute kidney injury: A systematic review meta-analysis. Int. Urol. Nephrol..

[56] Seibert F.S., Heringhaus A., Pagonas N., Rohn B., Bauer F., Trappe H.J., Landmesser U., Babel N., Westhoff T.H. (2021). Dickkopf-3 in the prediction of contrast media induced acute kidney injury. J. Nephrol..

[57] Roscigno G., Quintavalle C., Biondi-Zoccai G., De Micco F., Frati G., Affinito A., Nuzzo S., Condorelli G., Briguori C. (2021). Urinary Dickkopf-3 and Contrast-Associated Kidney Damage. J. Am. Coll. Cardiol..

[58] Enko D., Meinitzer A., Scherberich J.E., März W., Herrmann M., Artinger K., Rosenkranz A.R., Zitta S. (2021). Individual Uromodulin Serum Concentration Is Independent of Glomerular Filtration Rate in Healthy Kidney Donors. Clin. Chem. Lab. Med. CCLM.

[59] Garimella P.S., Biggs M.L., Katz R., Ix J.H., Bennett M.R., Devarajan P., Kestenbaum B.R., Siscovick D.S., Jensen M.K., Shlipak M.G. (2015). Urinary Uromodulin, Kidney Function, and Cardiovascular Disease in Elderly Adults. Kidney Int..

[60] Steubl D., Block M., Herbst V., Nockher W.A., Schlumberger W., Kemmner S., Bachmann Q., Angermann S., Wen M., Heemann U. (2019). Urinary Uromodulin Independently Predicts End-Stage Renal Disease and Rapid Kidney Function Decline in a Cohort of Chronic Kidney Disease Patients. Medicine.

[61] Vonbrunn E., Ebert N., Cordasic N., Amann K., Büttner A., Büttner-Herold M., Scherberich J.E., Daniel C. (2025). Serum Uromodulin as early marker for ischemic acute kidney injury and nephron loss: Association with kidney tissue distribution pattern. J. Transl. Med..

[62] Kaszynska A., Kepska-Dzilinska M., Karakulska-Prystupiuk E., Tomaszewska A., Basak G., Zorawski M., Drozak I., Jakubowska Z., Malyszko J. (2024). 226.9: Proenkephalin and DKK-3 levels and kidney function in patients after hematopoietic stem cell transplantation. Transplantation.

[63] Levey A.S., Eckardt K.U., Tsukamoto Y., Levin A., Coresh J., Rossert J., Zeeuw D.D., Hostetter T.H., Lameire N., Eknoyan G. (2005). Definition and classification of chronic kidney disease: A position statement from kidney disease: Improving global outcomes (KDIGO). Kidney Int..

